# Exploring the Role of Alcohol Metabolizing Genotypes in a 12-Week Clinical Trial of Naltrexone for Alcohol Use Disorder

**DOI:** 10.3390/biom11101495

**Published:** 2021-10-10

**Authors:** João M. Castaldelli-Maia, André Malbergier, Adriana B. P. de Oliveira, Ricardo A. Amaral, André B. Negrão, Priscila D. Gonçalves, Antonio Ventriglio, Domenico de Berardis, Juliana de Antonio, Isabela Firigato, Gilka J. F. Gattás, Fernanda de Toledo Gonçalves

**Affiliations:** 1 Interdisciplinary Group of Studies on Alcohol and Drugs (GREA), Institute of Psychiatry, Medical School, Universidade de São Paulo, São Paulo 05403-903, Brazil; andre.malbergier@hc.fm.usp.br (A.M.); abpileggi@uol.com.br (A.B.P.d.O.); ricardo.amaral@hc.fm.usp.br (R.A.A.); abnegrao@usp.br (A.B.N.); pri_dib@yahoo.com.br (P.D.G.); 2 Department of Neuroscience, Medical School, FMABC Health University Center, Santo André 09060-870, Brazil; 3 Department of Experimental and Clinical Medicine, University of Foggia, 71122 Foggia, Italy; a.ventriglio@libero.it; 4 Department of Neurosciences and Imaging, University “G. D’Annunzio” Chieti, 66100 Chieti, Italy; dodebera@aliceposta.it; 5 Departamento de Medicina Legal e Ética Médica, Medicina Social e do Trabalho, Instituto Oscar Freire, LIM-40, Medical School, Universidade de São Paulo, São Paulo 05405-150, Brazil; juitaju83@gmail.com (J.d.A.); isabela.firigato@usp.br (I.F.); gfgattas@usp.br (G.J.F.G.); fernandatoledo@gmail.com (F.d.T.G.)

**Keywords:** alcohol, naltrexone, genotyping, *ADH1B*, *ADH1C*, *ALDH2*

## Abstract

**Background:** The efficacy of naltrexone in the treatment of alcohol use disorder (AUD) has been associated with a set of variables not directly related with the expression of opioid receptors. All the variables have been found to be highly associated with AUD itself or more severe clinical levels of AUD. **Objectives:** Given the high association between alcohol metabolizing enzymes (AME) and the outcome of AUD, the present study aims to investigate the role of AME genotype variants in the treatment of AUD with naltrexone. **Methods:** We carried out a 12-week longitudinal clinical trial based on the treatment of AUD patients with naltrexone (N = 101), stratified by different alcohol metabolization genotypes. Genotyping was performed after the inclusion of the patients in the study, based on the individual presence of single nucleotide polymorphisms (SNPs) in the ADH (alcohol dehydrogenase)1B (ADH1B*2 and ADH1B*3), ADH1C (ADHC*1) and ALDH (aldehyde dehydrogenase) 2 (ALDH2*2) genes. The outcome of alcohol use has been monitored employing the timeline follow-back during the treatment. **Results:** The ADH1C*1 (Ile350Val, rs698) and ALDH2*2 (Glu504Lys, rs671) polymorphisms were associated with a better response to naltrexone treatment, whereas the ADH1B*3 (Arg370Cys, rs2066702) allelic variant showed a negative outcome. **Conclusions:** The present study explores a genomic setting for the treatment of AUD with naltrexone. According to our findings, the association between ADH1C*1 and ALDH2*2 variants and better outcomes suggests a successful treatment, whereas the ADH1B*3 mutated allele might lead to an unsuccessful treatment. Further studies should be performed to investigate the relationship between alcohol metabolizing genotypes, the family history of alcohol use disorders and the effect of naltrexone on the outcomes. Genotyping may be a valuable tool for precision-medicine and individualized approach, especially in the context of alcohol use disorders. The small number of subjects was the main limitation of the present study.

## 1. Introduction

Alcohol use disorder (AUD) is a major public health problem [1,2]. It has been included among the top-ten risk factors for days of activity lost [3]. AUD is a clinical syndrome defined by a set of diagnostic criteria [4] including the former criteria of alcohol dependence and abuse [5]. These criteria are proposed on the basis of genetic evidence [6]. In fact, an increased number of genetic variants for alcohol metabolization and heavy alcohol consumption have been proposed over the last decade [7,8] with particular attention to the role of genetic factors, that have been associated with the AUD outcome [6] and various clinical features [9]. Alcohol metabolizing enzyme (AME) genes, alcohol dehydrogenases (*ADH1B* and *ADH1C*) and aldehyde dehydrogenase (*ALDH2*), are genetic factors associated with the AUD outcome [6,10,11,12]. Several AME genes are located in the 4q23 region [13], which has been associated with numerous alcohol-use behaviors [14].

Regarding the AUD treatments, naltrexone is one of the main pharmacological therapies that was approved by the FDA (Food and Drug Administration) in 1994. This drug is an opioid antagonist on µ, δ and κ receptors that blocks the mesolimbic pathways and reduces heavy drinking and relapses [15]. Alcohol indirectly stimulates the endogenous pathways, releasing β-endorphins and enkephalins in the synaptic cleft, which play an excitatory role, mediated by dopamine-release in the *nucleus accumbens* and producing the pleasurable sensation associated with [16].

The efficacy of the naltrexone treatment has been associated with genetic variants, which are linked to some patterns of alcohol consumption [17,18]. In addition, variables such as pre-treatment drinking, family history of alcohol problems, male sex and high craving, also contribute to the success of the pharmacological AUD treatment [19]. Similarly, these variables have been highly associated with the syndrome itself and severe clinical levels of AUD [20,21,22,23].

Despite the results of genetic factors and further variables, largely replicated in psychiatric-genetic studies [6], the advanced genetic evidence on addiction has not been applied to effective treatments. Thus, the assessment of genetic characteristics may be crucial to the personalizing treatment and increase the effectiveness of the current pharmacological treatments for AUD. The present study aims to investigate the possible association between polymorphisms in AMEs polymorphism genes (ADH1B*2, ADH1B*3, ADH1C*1 and ALDH2*2) and the outcome of AUD treatment with naltrexone.

## 2. Methods

### 2.1. Participants

A total of 101 participants in the trial were computed on the basis of the sampling performed in previous similar studies [24,25,26]. Male patients who met the diagnostic criteria for alcohol use disorder according to the ICD (International Classification of Diseases) 10 years of age to older than 35 years of age were enrolled. People with psychotic or bipolar disorders, dementia and liver diseases were excluded from the present study. Psychiatric diagnoses were assessed through a psychiatric clinical examination made by a certified psychiatrist. Liver diseases were assessed through medical examination made by a certified physician, and laboratory tests were conducted. All the candidate patients went through psychiatric and clinical examination.

Enrollment was advertised in local media and participants were self-referred or referred by other outpatient services for the treatment of AUD. Recruitment occurred between February 2008 and September 2010 in the Institute of Psychiatry of Sao Paulo University Medical School, Brazil. First, all participants were interviewed by clinical psychiatrists (as stated), who evaluated the inclusion and exclusion criteria. After the enrollment, patients were assessed on the basis of their patterns of alcohol consumption through a structured questionnaire, measuring frequency and amount of alcohol use in the last month, as well as their sociodemographic data. All participants signed a written informed consent and the trial was approved by the Institutional Ethical Committee (CAPPesq number 0845/07).

### 2.2. Baseline Measures

#### 2.2.1. Genotyping

Blood samples (5 mL) were collected in EDTA (Ethylenediaminetetraacetic acid) and DNA (deoxyribonucleic acid), were isolated by the salting-out process, according to Miller et al. [27] and stored at −80 °C before the genotyping. Next, DNA samples were qualified and quantified using a NanoDrop™ 2000 spectrophotometer (Thermo Fisher Scientific, Waltham, MA, USA). An A260/A280 ratio between 1.8 and 2.2 was used to classify the samples as high genomic DNA quality.

Genotyping of ADH1B*2 (rs1229984), ADH1B*3 (rs2066702), ADH1C*1 (rs698) and ALDH2*2 (rs671) polymorphisms was determined by TaqMan^®^ SNP Genotyping Assays (Applied Biosystems, Foster City, CA, USA). The primers and probes were predesigned assays by Applied Biosystems, and genotyping was performed on the StepOnePlus™ instrumentation platform (Applied Biosystems, Foster City, CA, USA), according to the manufacturer’s recommendations. Description of the polymorphisms and their corresponding genes, including genomic coordinate, other names, amino acid change (for missense alterations), protein subunit encoded and TaqMan^®^ assays, is described in Table 1.

#### 2.2.2. Sociodemographic and Clinical Profile

We collected the following sociodemographic data: age (in years), level of education (up to middle school, high school, university), marital status (married or unmarried), religion (practicing or non-practicing), skin color (white or other), employment (formal job, informal job, not working) and housing status (own house or other). In addition, clinical data were collected as follows: daily smoking (current, former, never), age of onset of alcohol use (in years), mutual-help groups for alcohol use disorder (previous participation or not), outpatient treatment for alcohol use disorder (previous treatment or not), previous inpatient treatment for alcohol use disorders (never, emergency department and wards/communities), previous seizures (yes or no), delirium tremens, (yes or no), legal problems related to alcohol use (yes or no), use of illicit drugs in the last 30 days (yes or no), family history of alcohol use disorders (yes or no) and any alcohol-related diseases (yes or no).

### 2.3. Intervention

Patients were enrolled in a 12-week treatment with naltrexone. They were assessed by a clinical psychiatrist every week in the first month and fortnightly until the end of the study. A cut-off of less than three-times of the average normal levels of hepatic enzymes was adopted as a safety criterion for the prescription of oral naltrexone at 50 mg/day. The medication was dispensed after any psychiatric evaluation. All patients also attended the weekly psychotherapeutic groups. Weekly therapeutic groups were offered by psychologists trained in cognitive-behavioral therapy. Each week a topic related to alcohol use was addressed. Patients entered the group after the first medical evaluation and remained for 12 weeks.

### 2.4. Outcome Measures

The main variable for measuring the outcome was the number of daily alcohol doses and days of alcohol-use during the treatment period. The standard alcohol drink was 14 g of alcohol, defined as a 12-oz beer, 5-oz glass of wine or 1^1/2^-oz shot of spirits. Abstinence was defined as no days of alcohol use. Patients’ alcohol use was monitored by their clinical psychiatrist through a diary based on a timeline follow-back, as suggested by previous clinical studies on alcohol use disorders [28,29,30,31]. Psychiatric assessments also included a psycho-educational module about alcohol dependence and a personalized discussion about the impact of alcohol use on the lives of patients. Treatment retention was considered a secondary outcome of interest, measured by the count of the attendance days of each patient (maximum 84 days). All the outcomes were considered on an intention-to-treat basis, following previous clinical studies with patients with alcohol use disorders.

### 2.5. Statistical Methods

All analyses were performed using STATA statistical software, version 16 (Stata Corp., College Station, TX, USA). Initially, we calculated the proportion of each sociodemographic and clinical variable per abstinence status (i.e., no day of alcohol use during the treatment). Then, we carried out logistic and Poisson regression models for each category (abstinence) and count (days of alcohol use, days of 1 unit of alcohol use, days of 2 units of alcohol use, days of 3 units of alcohol use, days of 4 units of alcohol use and days of 5 of more units of alcohol use) outcomes, according to the polymorphism genotypes. All the association analyses were performed under a Dominant model due to the low frequency of the homozygous genotype (i.e., mutated (homozigous + heterozigous genotypes) vs. a wild type genotype as follows: ADH1B*2 vs. no-ADH1B*2, ADH1B*3 vs. no-ADH1B*3, ADH1C*1 vs. no-ADH1C*1 and ALDH2*2 vs. no-ALDH2*2) as the predictor. The sociodemographic and clinical variables which had significant differences for abstinence in the initial analysis were included as adjustment covariates in the multiple Poisson and logistic regression models. For these models, the Bonferroni correction was used to set significance at *p* < 0.0016 (0.05/32 models).

## 3. Results

Sociodemographic characteristics of the sample are presented in Table 2. A total of 101 individuals with ICD-10 alcohol use disorders were included in the present study. The mean age was 48.9 (95% CI = 47.2 to 50.5). A total of 51.5% of patients reported the lowest level of education (up to middle school), 56.4% of subjects were married, 76.2% of individuals presented with white skin color, 59.4% of participants practiced religion, 72.3% of patients were employed (27.7% formally and 44.6% informally) and 77.8% of subjects were living in their own houses. After a 12-week naltrexone treatment, 37 individuals were abstinent (36.6%). There were no significant sociodemographic differences between abstinent and non-abstinent groups.

Clinical characteristics of the sample at baseline are presented in Table 3. The mean age at onset of alcohol use was 16 years old. Out of those who were ever in an alcohol treatment, 55.4% (56) of them reported a previous outpatient treatment, of which 49 (87.5%) reported at least one inpatient treatment, 30 individuals were admitted in wards or communities, 43 of them attended to mutual-help groups and 19 of patients required emergency support at least once. There was a statistically significant (*p* < 0.05) difference in the frequency distribution of the outpatient treatment between the non-abstinent (61.9%) and abstinent (45.9%) groups.

Regarding the alcohol-related issues, 38 patients reported a previous delirium tremens episode and legal problems, 27.7% of individuals reported seizures and 32.7% reported an alcohol-related disease (e.g., hepatitis and neuropathy), with significant differences in the frequency distribution between the non-abstinent (35.9%) and abstinent (27.1%) groups (*p* < 0.05). Around 5% of the sample reported other drug-use in the last 30 days before the study. Furthermore, 67.3% were current smokers. Almost 90% of individuals had a family history of alcohol use disorders that showed a statistically significant (*p* < 0.001) difference between the non-abstinent (92.2%) and abstinent (78.4%) groups. The prevalence of mutated genotypes (homozygous + heterozygous) was 43.6% for the ADH1C*1 polymorphism, about 5% for both ADH1B*2 and ADH1B*3 and 4% for ALDH2*2. None of these mutated genotypes were correlated with family history of alcohol use disorders in our sample.

Concerning the outcome measures, the mean number of days for drinking and retention were 8.6 (95% CI = 5.6 to 11.6) and 67.0 (95% CI = 61.6 to 72.3), respectively. Among the drinking doses per day of use, they ranked: heavy drinking (5 or more doses) with a mean of 2.3 days of use (95% CI = 1.2 to 3.5), 2 doses (mean = 2.2, 95% CI = 1.3 to 3.2) and 1 dose (mean = 1.9, 95% CI = 1.0 to 2.9). Subjects preferred 1 (34.5%), 2 (36.3%), 3 (29.7%), 4 (30.8%) or 5 or more drinks (34.1%) in the days of use and 37% did not drink during the entire period.

Table 4 presents the correlation between outcomes and genotypes ADH1B*2, ADH1B*3, ADH1C*1 and ALDH2*2 through adjusted Poisson and logistic regression models. No significant correlation was found for the ADH1B*2 genotypes. Patients with mutated ADH1B*3 reported more days of alcohol use (coef = 0.57, 95% CI = 0.34 to 0.80, *p* < 0.001), 1 drink (coef = 0.74, 95% CI = 0.29 to 1.19, *p* = 0.001), 2 drinks (coef = 0.86, 95% CI = 0.48 to 1.23, *p* < 0.001) and 5 drinks or more (coef = 0.61, 95% CI = 0.11 to 1.01, *p* = 0.015). On the other hand, patients that presented with the mutated ADH1C*1 reported less days of alcohol use (coef = −0.31, 95% CI = −0.45 to −0.16, *p* < 0.001), 1 drink (coef = −0.91, 95% CI = −1.25 to −0.57, *p* < 0.001) and 2 drinks (coef = −0.69, 95% CI = −1.00 to −0.39, *p* < 0.001). The days of alcohol use were also diminutive among the patients who carried mutated ALDH2*2 (aOR = −0.91, 95% CI = −1.15 to −0.45, *p* < 0.001).

## 4. Discussion

This study aimed to investigate the role of genotype variants of AMEs genes in the treatment outcome with naltrexone. The ADH1C*1 and ALDH2*2 polymorphic genotypes were found to be associated with better response for treatment, whereas ADH1B*3 variant genotypes were found to be less successful. Our findings support the use of naltrexone in those patients who might be less susceptible to alcohol dependence (i.e., ADH1C*1 and ALDH2*2), wherein these polymorphisms have been also related to the AUD protection [6,10,11,12,32]. Other medications (e.g., acamprosate or disulfiram) could have better results in those with the ADH1B*3 variant genotype, and should be tested in further studies.

Genetic epidemiological studies indicated that the higher protective effects for AUD were found among individuals who presented with the ADH1B*2 and ALDH2*2 variant genotypes [33,34,35]. Other variations in *ADH* and *ALDH* genes may also affect the risk of alcohol dependence and abuse, as ADH1C*1 and ADH1B* [32]. Thus, our findings indicate a putative protective effect of ALDH2*2 and ADH1C*1 for alcohol behaviors and treatment response, while the ADH1B*3 variant genotypes might contribute to a worse clinical outcome. Based on previous findings of protective effects of all these variants on alcohol use behaviors [6,10,11,12,32], we speculate that these individuals should also have lower post-treatment relapse levels compared to those without these variants.

There is a lack of evidence on the inter-relation among alcohol metabolizing genotypes with the naltrexone outcome treatment. Ray et al. [36] explored the role of the alcohol enzyme metabolizing genotypes on the effect of naltrexone in alcohol intoxication and craving. The study was a double-blinded, randomized, placebo-controlled laboratory trial of naltrexone versus placebo, reporting no significant effect of *ALDH2* or *ADH1B* polymorphism genotypes on the outcomes of treatment.

Besides the AMEs polymorphisms genes, the naltrexone success treatment also is related to other clinical and biological characteristics. The systematic review conducted by Garbutt et al. [19] showed that naltrexone efficacy has been associated with pre-treatment drinking, family history of alcohol problems, polymorphism of the μ-opioid receptor gene, male sex and high craving. Bujarski et al. [37] also reported that a family history of alcohol, associated with a pattern of the alcohol metabolizing genotype, might impact negatively on alcohol behaviors. In the present study, outpatient treatment, alcohol-related diseases and family history of alcohol use disorders were also associated with the better treatment response of naltrexone.

The impact of our findings on further genomic evidence for the treatment of alcohol use disorders is potentially interesting. Genotyping has been considered as a possible tool for precision medicine, with a special focus in the area of mental health [38,39,40,41]. Even if many studies supported the genotyping *OPRM1* in the treatment with naltrexone [42,43,44], our data would suggest genotyping for *ADH1B*, *ADH1C* and *ALDH2* before the treatment. Further studies should be carried out to assess the potential role of each genotype in the treatment outcome.

Limitations may include a small sample size compared to previous studies [24,25,26]. Moreover, as expected for predominantly Caucasian samples [45,46], we found just a small number of individuals with the variant genotypes ADH1B*2, ADH1B*3 and ALDH2*2. Unfortunately, we did not include a control group taking other pharmacological interventions for alcohol use disorder, such as acamprosate, disulfiram, gabapentin, topiramate, or a placebo [15]. In addition, we only included male individuals, and the alcohol-use clinical outcome assessment was based on self-report only; this was based on methodology suggested in recent randomized clinical prospective studies [47,48,49,50]. Interestingly, this study genotyped 100 individuals with AUD for four genotype variants of interest [14,51,52], as well as analyzed their association with the clinical assessment measures during a standardized 12-week treatment, based on an FDA approved intervention [53] and an intention-to-treat analysis [54]. This was the first study reporting on the differential role of *ADH1B* variant genotypes for the treatment of AUD. In fact, there is a lack of genomic studies in developing countries, especially investigating alcohol metabolizing enzyme genotypes in Caucasian samples. The majority of these studies have been conducted among Asian ethnicities or Asian descendants [55,56,57,58]. However, we were not able to find any significant genotypic association with abstinence in this trial.

## 5. Conclusions

This study added evidence to the genetic factors associated with the AUD treatment with naltrexone (Graphic Abstract). The ADH1C*1 and ALDH2*2 variant genotypes seemed to be facilitators, whereas the ADH1B*3 variant genotypes were not associated with a positive outcome of treatment. Further studies should investigate the relationship among alcohol metabolizing genotypes, family history of alcohol use disorders and the effects of naltrexone. Genotyping might be a valuable tool for precision medicine, especially in the context of alcohol use disorders.

## Figures and Tables

**Table 1:** Description of the polymorphisms and their corresponding genes, including genomic coordinate, other names, amino acid change (for missense alterations), protein subunit encoded and TaqMan^®^ assays code.

Genes	Polymorphisms (rs)	Genomic Coordinate	Other Names	Transition	Protein Subunit	TaqMan^®^ Assays
*ADH1B*	ADH1B*2 (rs1229984)	Chr.4: 99318162	ADH2*2	G→A Arg48His	β2-ADH	C___2688467_20
*ADH1B*	ADH1B*3 (rs2066702)	Chr.4: 99307860	ADH2*3	C→T Arg370Cys	β3-ADH	C__11941896_20
*ADH1C*	ADH1C*1 (rs698)	Chr.4: 99339632	ADH3*1	A→G Ile350Val	γ1-ADH	C__26457410_10
*ALDH2*	ALDH2*2 (rs671)	Chr.12: 111803962	ALDH2*Lys504	A→G Glu504Lys	ALDH2*2	C__11703892_10

*ADH*: alcohol dehydrogenase; *ALDH*: aldehyde dehydrogenase; Chr: chromosome; Lys: lysine; Arg: arginine; His: histidine; Cys: cysteine; Ile: isoleucine; Val: valine; Glu: glutamic acid; G: guanine; A: adenine; C: cytosine; T: thymine.

**Table 2:** Sociodemographic data of the sample (after a 12-week naltrexone treatment).

Sociodemographic Data	Total Sample (*n* = 101)	Non-Abstinent (*n* = 64)	Abstinent (*n* = 37)
*Age (years), mean (SE)*	48.9 (0.80)	49.4 (1.14)	47.9 (1.08)
*Level of education*			
*Up to middle school, n (%)*	52 (51.5)	31 (48.4)	21 (56.8)
*High school, n (%)*	36 (35.6)	24 (37.5)	12 (32.4)
*University, n (%)*	13 (12.9)	9 (14.1)	4 (10.8)
*Marital status, ever married, n (%)*	57 (56.4)	35 (54.7)	22 (59.4)
*Religion, practicing, n (%)*	60 (59.4)	38 (59.3)	22 (59.4)
*Ethnic group, white, n (%)*	77 (76.2)	49 (76.5)	28 (75.6)
*Occupation*			
*Formal job, n (%)*	28 (27.7)	16 (25.0)	12 (32.4)
*Informal job, n (%)*	45 (44.6)	29 (45.3)	16 (43.2)
*Not working, n (%)*	28 (27.7)	19 (29.7)	9 (24.3)
*Housing status, own house, n (%)*	77 (77.78)	48 (77.4)	29 (78.4)

Note: Regression models comparing the outcome of male dependents after receiving a 12-week naltrexone treatment: no significant differences.

**Table 3:** Baseline clinical characteristics and descriptive analysis.

Clinical Characteristics	Total Sample (*n* = 101)	Non-Abstinent (*n* = 63)	Abstinent (*n* = 37)
*Age at onset of alcohol use (years), mean (SE)*	16.5 (0.44)	16.5 (0.51)	16.4 (0.82)
*Previous treatment*			
*Mutual-help groups, n (%)*	43 (42.6)	31 (49.2)	12 (32.4)
*Outpatient treatment, n (%)*	56 (55.4)	39 (61.9)	17 (45.9) *
*Inpatient treatment, n (%)*	49 (48.5)	35 (54.7)	14 (37.8)
*Emergency department, n (%)*	19 (18.8)	14 (22.2)	5 (13.5)
*Wards/communities, n (%)*	30 (29.7)	21 (32.8)	9 (24.3)
*Never, n (%)*	45 (44.6)	25 (39.1)	20 (54.1)
*Seizures, n (%)*	28 (27.7)	17 (26.6)	11 (29.7)
*Delirium tremens, n (%)*	38 (37.6)	20 (31.3)	18 (48.7)
*Legal problems, n (%)*	38 (37.6)	24 (37.5)	14 (37.8)
*Drug use (30-days), n (%)*	5 (5.0)	4 (6.3)	1 (2.7)
*Alcohol-related diseases, n (%)*	33 (32.7)	23 (35.9)	10 (27.1) *
*Family history of alcohol use Disorders, n (%)*	88 (87.1)	59 (92.2)	29 (78.4) **
*Daily smoking*			
*Current, n (%)*	68 (67.3)	47 (73.4)	21 (56.8)
*Former, n (%)*	21 (20.8)	11 (17.2)	10 (27.0)
*Never, n (%)*	12 (11.9)	6 (9.4)	6 (16.2)
*Alcohol metabolization genotypes*			
ADH1B*2, n (%)	5 (5.0)	4 (5.1)	1 (4.6)
ADH1B*3, n (%)	5 (5.0)	3 (3.9)	2 (9.1)
ADH1C*1, n (%)	44 (43.6)	28 (44.4)	16 (43.2)
ALDH2*2, n (%)	4 (4.0)	2 (2.6)	2 (9.1)

Note: Regression models comparing male dependents outcome after receiving a 12-week naltrexone treatment. Treatment: * *p* < 0.05; ** *p* < 0.001.

**Table 4:** Correlation between clinical outcomes and genotyping (ADH1B*2, ADH1B*3, ADH1C*1 and ALDH2*2) through regression models adjusted for previous outpatient treatment, family history of alcohol problems and alcohol-related diseases.

Variables	ADH1B*2				ADH1B*3				ADH1C*1				ALDH2*2			
	Coef.	95% CI		*p*	Coef.	95% CI		*p*	Coef.	95% CI		*p*	Coef.	95% CI		*p*
		Lower	Upper			Lower	Upper			Lower	Upper			Lower	Upper	
*Days of alcohol use ^a^*	−0.14	−0.46	0.16	0.353	**0.57**	**0.34**	**0.80**	**<0.001**	**−0.31**	**−0.45**	**−0.16**	**<0.001**	**−0.91**	**−1.15**	**−0.45**	**<0.001**
*Days of alcohol use (1 unit) ^a^*	0.36	−0.22	0.96	0.223	**0.74**	**0.29**	**1.19**	**0.001**	**−0.91**	**−1.25**	**−0.57**	**<0.001**	−0.79	−1.80	0.21	0.121
*Days of alcohol use (2 units) ^a^*	−0.05	−0.61	0.50	0.854	**0.86**	**0.48**	**1.23**	**<0.001**	**−0.69**	**−1.00**	**−0.39**	**<0.001**	−0.98	−1.97	0.01	0.052
*Days of alcohol use (3 units) ^a^*	−0.01	−0.75	0.73	0.984	−0.88	−2.04	0.28	0.137	−0.48	−0.96	−0.01	0.047	−0.32	−1.14	0.49	0.438
*Days of alcohol use (4 units) ^a^*	−0.53	−1.55	0.53	0.303	0.24	−0.51	1.00	0.53	0.47	0.07	0.87	0.020	−1.26	−3.05	0.51	0.164
*Days of alcohol use (5+ units) ^a^*	−1.06	−2.06	−0.06	0.038	**0.61**	**0.12**	**1.11**	**0.015**	0.14	−0.12	0.41	0.287	−13.51	−734.19	707.16	0.971
*Abstinence ^b^*	0.32	0.03	3.21	0.335	0.95	0.14	6.47	0.962	1.23	0.51	2.97	0.638	4.05	0.47	34.39	0.200

Note: Bold–significant correlations after Bonferroni correction (*p* < 0.0016); *^a^* Poisson Regression model; *^b^* Logistic Regression model.

## Data Availability

The data that support the findings of this study are available from *Programa Interdisciplinar de Estudos de Álcool e Drogas* (https://www.grea.org.br, accessed on 3 October 2021) but restrictions apply to the availability of these data, which were used under license for the current study, and so are not publicly available. Data are however available from the authors upon reasonable request and with permission of *Programa Interdisciplinar de Estudos de Álcool e Drogas* (https://www.grea.org.br, accessed on 3 October 2021).
